# The roles of base excision repair enzyme OGG1 in gene expression

**DOI:** 10.1007/s00018-018-2887-8

**Published:** 2018-07-24

**Authors:** Ruoxi Wang, Wenjing Hao, Lang Pan, Istvan Boldogh, Xueqing Ba

**Affiliations:** 1 Key Laboratory of Molecular Epigenetics of Ministry of Education, Institute of Genetics and Cytology, Northeast Normal University, 5268 Renmin Street, Changchun, 130024 Jilin China; 2 School of Life Science, Northeast Normal University, Changchun, 130024 Jilin China; 3 Department of Physiology, Xiangya Medicine School in Central South University, Changsha, 410078 Hunan China; 4 Department of Microbiology and Immunology, University of Texas Medical Branch at Galveston, Galveston, TX 77555 USA; 5 Sealy Center for Molecular Medicine, University of Texas Medical Branch at Galveston, Galveston, TX 77555 USA

**Keywords:** Transcription modulation, Epigenetic, DNA methylation, Post-repair signaling

## Abstract

Modifications of DNA strands and nucleobases—both induced and accidental—are associated with unfavorable consequences including loss or gain in genetic information and mutations. Therefore, DNA repair proteins have essential roles in keeping genome fidelity. Recently, mounting evidence supports that 8-oxoguanine (8-oxoG), one of the most abundant genomic base modifications generated by reactive oxygen and nitrogen species, along with its cognate repair protein 8-oxoguanine DNA glycosylase1 (OGG1), has distinct roles in gene expression through transcription modulation or signal transduction. Binding to 8-oxoG located in gene regulatory regions, OGG1 acts as a transcription modulator, which can control transcription factor homing, induce allosteric transition of G-quadruplex structure, or recruit chromatin remodelers. In addition, post-repair complex formed between OGG1 and its repair product-free 8-oxoG increases the levels of active small GTPases and induces downstream signaling cascades to trigger gene expressions. The present review discusses how cells exploit damaged guanine base(s) and the authentic repair protein to orchestrate a profile of various transcriptomes in redox-regulated biological processes.

## Introduction

DNA base damage (including deamination, oxidation, or alkylation) is repaired through base excision repair (BER) pathway, which is highly conserved in pro- and eukaryotes [1]. To date, BER is believed to be the simplest, thoroughly characterized process among all DNA repair pathways [2, 3]. BER is initiated by mono- or bi-functional DNA glycosylase(s). After base release, apurinic/apyrimidinic (AP) site processing, nucleotide incorporation, and nick sealing are successively carried out by AP endonuclease1 (APE1), DNA polymerase β, and DNA ligase in a “hand-off” model [2, 4, 5].

Among four DNA bases, guanine has the lowest oxidation potential [6, 7]; thus, its oxidation product 7,8-dihydro-8-oxoguanine (8-oxoG) is the most predominant oxidative damage and taken as a biomarker of oxidative stress [6–9]. 8-Oxoguanine DNA glycosylase1 (OGG1), a functional analog of *Escherichia coli* protein MutM/Fpg, is tailored to specially remove 8-oxoG and its open-ring product 2,6-diamino-4-hydroxy-5-formamidopyrimidine (FapyG) from DNA duplex [10–13]. 8-OxoG is pre-mutagenic, because it may pair with adenine instead of cytosine, resulting in a G:C-to-T:A transversion during DNA replication [14, 15]. Despite the vulnerability of guanine and the mutagenicity of 8-oxoG, the compositional pattern of the human genome shows a high level of heterogeneity, and the density of genes is much greater in the GC-rich regions [16, 17]. In addition, 72% human gene promoters are guanine-rich [18]. Intriguingly, the transcriptional activity of genes is positively correlated with the GC content in gene regulatory regions [16, 17]. The genes prone to be actively transcribed are densely distributed in high GC-content regions, whereas those in a tissue-dependent or developmentally regulated manner are usually sparsely distributed in GC-poor regions. The correlation of the GC content and the distribution pattern of genes imply that guanine oxidation and its cognate enzyme OGG1 have the potential to be exploited in gene expression.

Recently, increasing evidence is calling attention to the non-repair functions of BER enzymes [19–23]. This review aims to discuss the significance of 8-oxoG as an epigenetic mark and stimuli-driven roles of OGG1 in gene expression modulation.

## Pre-base-excision role of OGG1 in transcription regulation

It has been well established that the intracellular reactive oxygen species (ROS) act as signaling molecules [24]. The putative mechanism of cell responses to ROS has long been attributed to thiol modifications on cysteine (Cys) residue(s) of the proteins that play key roles in intracellular signal pathways [25–27]. Attention has been focused on the effect of ROS on the redox status of reactive Cys residues located within the DNA-binding domain of transcription factors (TFs). The redox status of Cys may control the transcriptional activity of the TFs, including Nuclear Factor kappa B (NF-κB), activator protein 1 (AP-1), transcriptional activator Myb (Myb), cyclic adenosine 3,5-monophosphate response element-binding protein (CREB), early growth response protein 1 (Egr-1), hypoxia-inducible factor 1 alpha (HIF-1α), and tumor protein p53 (TP53) [28–32]. Recent research described a unique model how ROS influence transcription activation, which involves guanine oxidation in promoter regions and a pre-base-excision role of OGG1 in transcription.

The transcriptional activation of pro-inflammatory genes is mostly regulated by ROS-mediated signaling. However, pro-inflammatory genes usually have high GC-content promoters that are readily to be oxidized under oxidative stress. In response to stimuli, mRNA levels of TNF-α, CXCL1, CXCL2, CCL20, and IL-1β were rapidly (within 30 min) and robustly upregulated in human HEK293 cells, murine MLE-12 cells, and mouse lung. The induction of pro-inflammatory genes was significantly diminished due to OGG1 deficiency [33–35]. Inflammatory stimuli increase intracellular ROS, and consequently, the level of guanine lesions. How OGG1 facilitates the transcription from GC-rich promoters may be readily explained as that ROS damage high G-content promoters, and the repair of guanine lesions secures promoter integrity and ensures the recognition of *trans* factors to their *cis* elements. However, the burst of pro-inflammatory gene expression is coincided with the summits of intracellular ROS level, as well as genomic 8-oxoG content in gene regulatory regions [33–35]. Guanine lesions left unrepaired were interpreted because of cysteine-based enzymatic inactivation of OGG1 under oxidative stress, and OGG1 can regain its repair activity after redox balance reestablished [34, 36–38]. Chromatin immunoprecipitation (ChIP) and molecular biological assays further showed that, along with the increase in 8-oxoG level in promoters, OGG1 binds to the substrates without removal of the latter. The binding of enzymatically inactive OGG1 at promoters was followed by the assembly of transcriptional machinery. The interaction of OGG1 with sequence-specific TFs including NF-κB and specificity protein 1 (Sp1), general TFs such as TF IID, and phosphorylated RNA polymerase II, was induced upon the exposure of cells to the inflammatory cytokine TNF-α. However, these interactions were prevented by ROS scavenger [33]. ChIP assay showed a decreased enrichment of NF-κB on promoters after OGG1 depletion, which supports the role of OGG1 in the recruitment of components of transcriptional machinery [33, 34].

**Fig. 1:**
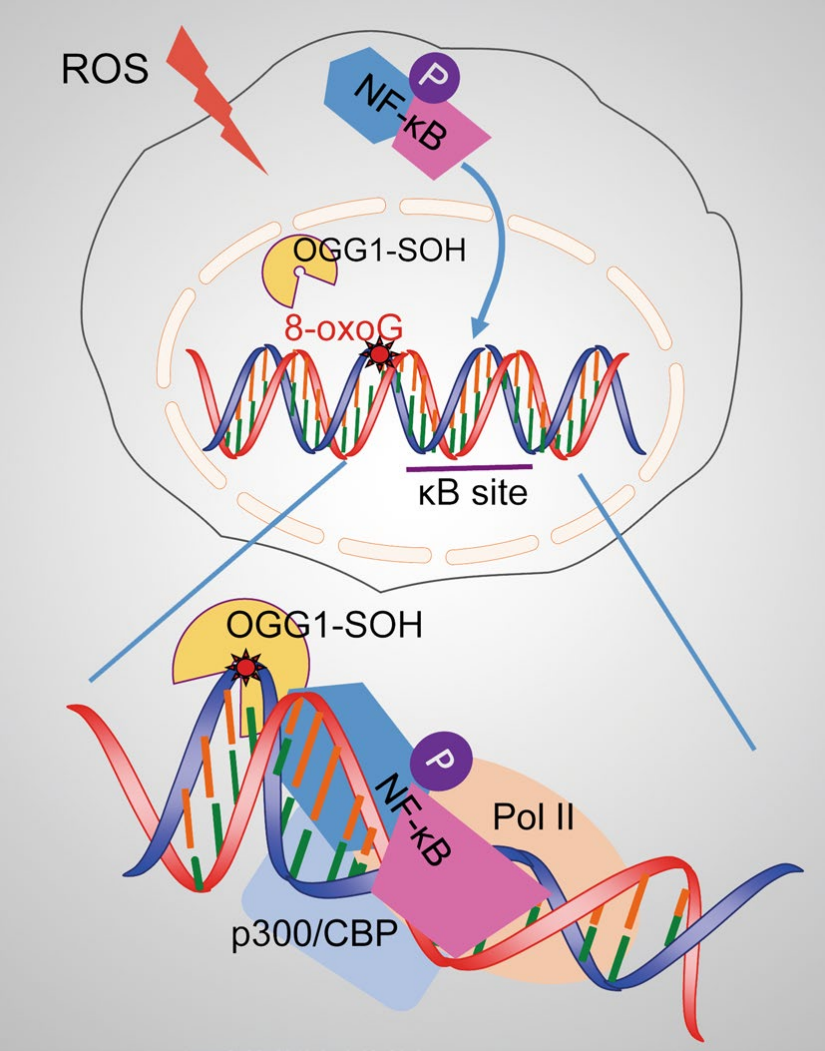
OGG1 modulates the transcription of NF-κB target genes. Enzymatically inactive OGG1 (OGG1-SOH) by ROS binds to 8-oxoG located in gene regulatory regions and induces allosteric alteration of DNA, which facilitates NF-κB occupancy and the assembly of the transcriptional initiation complex

NF-κB, a master regulator of gene expression, has been shown to be regulated by ROS that are induced by various inflammatory stimuli including cytokines/chemokines and infectious agents [39–41]. The mechanism by which DNA repair enzyme OGG1 engages with its genomic substrates is well established; however, the effect of OGG1-substrate engagement on NF- κB recruitment and its transcription activation has not been fully elucidated. The role of guanine lesions located within binding motifs of TFs (such as NF-κB, Sp1, AP-1, and CREB1) with or without the presence of OGG1 has been investigated by utilizing synthetic DNA and electrophoretic mobility shift assays (EMSAs). In the lack of OGG1, the effects of replacement of individual guanines for 8-oxoG in motifs of TFs resulted in the altered TF binding, yet the conclusions drawn from these studies were controversial [42–45]. Recently, it was demonstrated that recombinant OGG1 or the nuclear extract from OGG1-expressing cells enhanced the binding of NF-κB to DNA with 8-oxoG located several nucleotides upstream from the motif, whereas, 8-oxoG being within or closely proximal to NF-κB-binding site decreased its occupancy, which may involve either 8-oxoG itself or OGG1’s shielding effect [34, 35]. Wang et al. analyzed the surrounding sequences (10 bp upstream and downstream from the motif) of total 70 functional human κB sites. Their data revealed that the frequencies of guanine existence at − 8 and − 10 bases away from κB motif are more than 40%, notably higher than other positions [46], implying that the correctly positioned guanine oxidation may favor homing of TFs to sites through interaction with the cognate repair enzyme. Given that BER involves a strand break intermediate [44], a model how OGG1 modulates the transcription of NF-κB-targeting cytokine/chemokine genes may be described as follows. Timely oxidative inactivation of OGG1 prevents the burst of strand break generation in G cluster-containing promoters. Despite the compromised catalytic activity, binding of OGG1 to its substrate still possesses the capability to introduce a sharp (~ 70°) bend of the DNA duplex in the pre-lesion-excision step of BER [47–49], which induces allosteric change of DNA in chromatin context and creates a specific interface allowing the prompt recognition of motifs by NF-κB and then the assembly of the transcriptional initiation complex [50] (Fig. 1).

Pro-inflammatory cytokines/chemokines represent a typical class of innate immune response genes that can be immediately upregulated along with the burst of intracellular ROS. Evidence is showing that, in a timely view, guanine’s oxidation product is not only a lesion to be repaired but serves as a ligand for OGG1, and together, they play a role in the recruitment of TFs and the assembly of transcriptional machinery [33–35], to assure a prompt launch of the immediately responsive transcriptome. Studies are suggesting that guanine in DNA duplex is an ROS sensor, and OGG1 is a coordinator, to regulate the transcription from ROS-responding genes in the biological processes such as immune response [21, 51].

## OGG1-BER enzymatic activity-dependent promoter activation

**Fig. 2:**
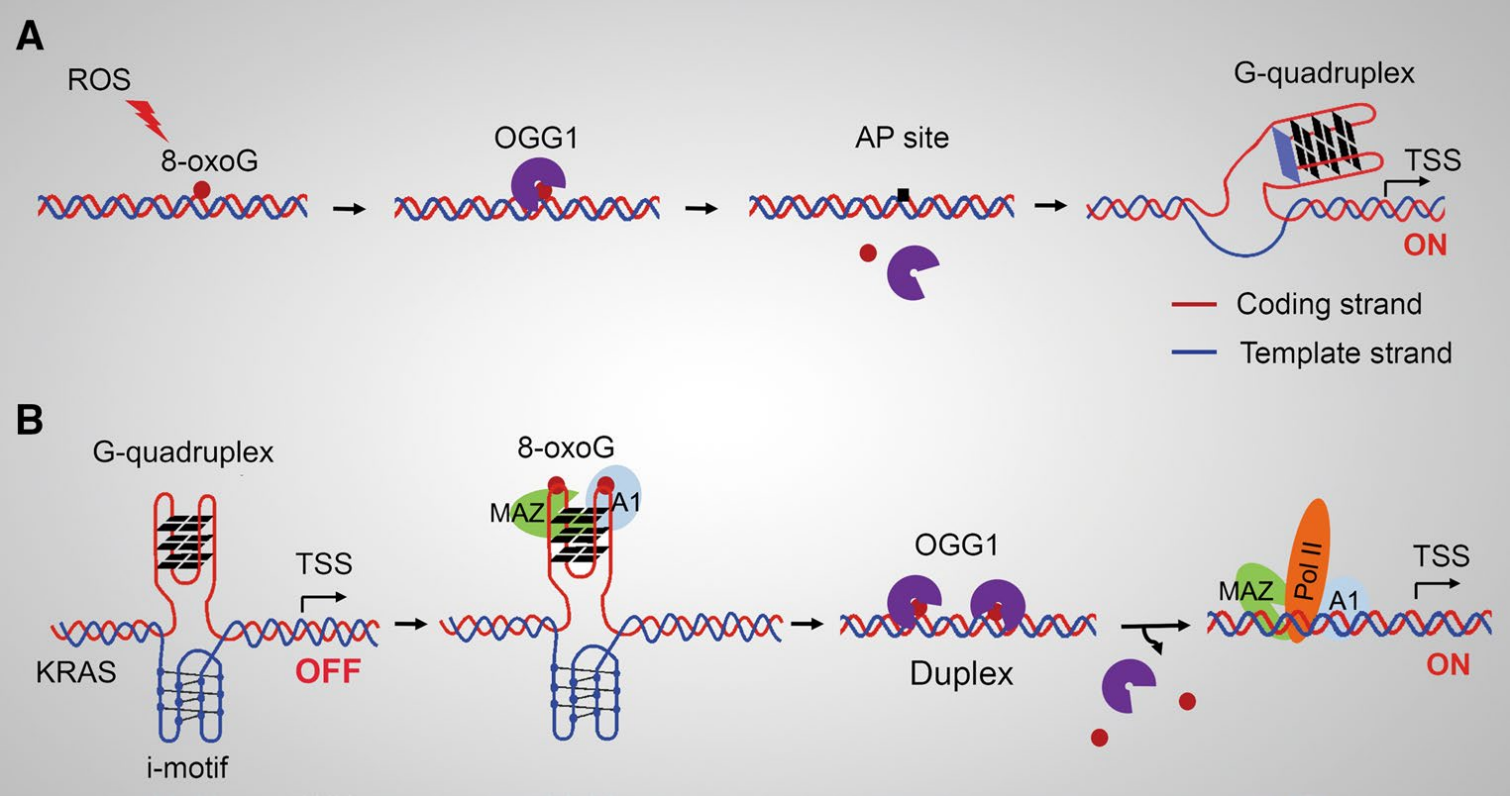
Roles of OGG1 in modulating transcription from G-quadruplex-containing promoters. **a** OGG1-initiated 8-oxoG removal from the coding stand of promoter allows transformation of PQS into G-quadruplex and transcriptional activation. **b** 8-oxoG in the *KRAS* PQS facilitates the reconstitution of the double helix. OGG1 excises 8-oxoG from PQS in double helix and thereby facilitates the binding of nuclear factors to their cognate sequences, resulting in activation of transcription. *PQS* potential G-quadruplex-forming sequences

Studies also depicted mechanisms by which OGG1 plays key roles in transcriptional activation depending on its BER activity and the removal of its substrate. Dr. Gillespie’s study documented that, in hypoxia, mitochondria-generated ROS stimulate the accumulation of hypoxic gene transcriptional regulator hypoxia-inducible factor-1 (HIF-1) [52], and cause oxidative base modifications in hypoxic response elements (HREs) of hypoxia-inducible genes [53]. When the hypoxia ROS-induced base modifications are prevented or OGG1 expression is inhibited, HIF-1 fails to associate with the HRE in the vascular endothelial growth factor (*VEGF)* promoter and gene expression at mRNA level is blunted [53]. The precise molecular mechanism, by which 8-oxoG formed in promoter upregulates VEGF expression, was dissected by Dr. Burrows’ group. When 8-oxoG is formed in guanine-rich, potential G-quadruplex-forming sequences (PQS) in coding strand of the promoter, OGG1 yields an AP site. The AP site enables melting of the duplex to unmask the PQS, adopting a G-quadruplex fold (G4 structure/motif) that has regulatory role in transcription activation (Fig. 2a) [54]. APE1 binds to, but inefficiently cleaves AP site, inducing transcription activation of *VEGF* or *endonuclease III*-*like protein 1* (*NTHL1*) genes, most likely with the aid of other activating factors [55]. In this hypoxia-induced transcription activation model, function of OGG1 is manifested at a post-lesion-excision stage. In this scenario, in response to hypoxia-induced ROS, Hif-1 accumulation is primary for VEGF expression; thus, it is likely that the resume of glycosylase activity of OGG1 due to the restoration of physiological cellular redox status [36, 37] is the key for adoption of the *cis* element G-quadruplex fold, which is temporally coordinated with the accumulation of the *trans* factor Hif-1. The analyses of PQSs recently were extended to promoters of DNA repair genes, and results showed that PQSs exist at a high density. Gene expression increases when the PQS is in the coding strand, whereas decrease when the PQS is in the template strand [56]. However, 8-oxoG in the template strand of the promoter is not repaired by BER, but primarily by transcription-coupled (TC)-nucleotide excision repair (NER) [57]. G4 formation has been observed in synthetic oligonucleotide sequences derived from the human genome, particularly those from gene promoters and telomeres. Computational predictions suggest that more than 300,000 sequence motifs in the human genome have the potential to form a G4 structure [58]. While the fact that G4 structure forms in promoter regions in vivo and whether its stabilization constitutes a layer of epigenetic gene expression regulation requires further experimental support, OGG1’s role in gene transcription modulation through G4 structure formation needs to be unscrambled in biologically relevant contexts.

Proto-oncogenes in human genome [such as MYC and Kirsten Ras (K-RAS)] have PQS in their promoters, whose activation is influenced by oxidative stress [59]. A recent study presented another role of OGG1 in G-quadruplex-based transcription regulation, during which OGG1 behaves as a bona fide BER enzyme. In the promoter of the KRAS oncogene, there is a G-rich region, which able to fold into a G-quadruplex structure (G4 motif). This unusual DNA conformation is recognized by nuclear proteins including MYC-associated zinc-finger protein (MAZ) and heterogeneous nuclear ribonucleoprotein A1 (hnRNP A1) [59, 60]. MAZ recognizes runs of guanines, unfolds the G-quadruplex, and leads to the transformation of G-quadruplex into duplexes [60]. Guanine oxidation is shown to be higher in a sequence able to fold into G4 motif compared with other G-rich regions [59]. The incorporation of 8-oxoG in a G-tetrad enhances the recruitment of MAZ and hnRNP A1, and destabilizes the G-quadruplex [59]. OGG1 is recruited to the KRAS G4 motif region that carries 8-oxoG more than non-G4 regions when the cells are treated with hydrogen peroxide (H_2_O_2_) or nuclear factor (erythroid-derived 2)-like 2 (NRF2) inhibitor that causes increased cellular ROS [59]. In this scenario, 8-oxoG itself first plays a positive role in promoting the recruitment of MAZ and hnRNP A1, whose first role is to unfold the inhibitory G-quadruplex into duplex. And then, 8-oxoG becomes negative due to its impediment to the sequence recognition of MAZ, hnRNP A1 (whose roles are as TFs at this stage), as well as other components of transcriptional machinery; thus, OGG1, maybe plus APE1, removes the obstacle, augmenting the promoter occupation of the transcriptional machinery (Fig. 2b).

**Fig. 3:**
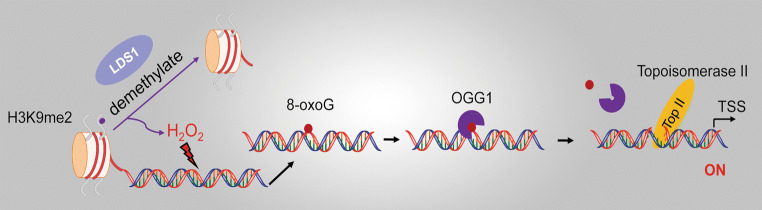
OGG1-BER-generated strand break activates transcription. The enzymatic activity of the lysine-specific demethylase LSD1 gives rise to a localized generation of H_2_O_2_, which oxidize G to 8-oxoG. Subsequently, OGG1-initiated base excision creates strand break, which serves as an entry for Top IIβ, triggering DNA conformational changes to accommodate the transcription initiation complex. *LSD* lysine-specific demethylase1, *Top IIβ* topoisomerase II beta

A previous study also documented that the productive transcription is achieved upon the formation of the strand break generated through OGG1-BER [61]. Estrogen (E2) treatment induced estrogen receptor alpha (ERα) binding to estrogen-responsive DNA elements (EREs), as well as loop formation between the promoter of anti-apoptotic gene *bcl*-*2* and the ERE enhancer. In parallel, the lysine-specific demethylase1 (LSD1)-triggered demethylation of K3K9me2 at promoter and enhancer sites resulted in the production of ROS. Estrogen-caused rapid genomic accumulation of 8-oxoG was tightly linked to LSD1 activation, because it was inhibited by monoamine oxidase inhibitor pargyline or LSD1 knockdown. Systematic analysis of chromatin downstream from *bcl*-*2* EREs revealed that OGG1 and topoisomerase IIβ accumulated preferentially at the promoter and ERE sites, which was dependent on E2-induced activation of LSD1. Removal of the oxidized guanines by OGG1 generates transient nicks that function as the entry points for topoisomerase IIβ, triggering DNA conformational change to accommodate the transcription initiation complex to achieve transcription [61] (Fig. 3). In vitro studies demonstrated that OGG1 has glycosylase and AP-lyase activity, the latter cleaving the phosphodiester backbone at 3′ of the damaged base [42, 43]. However, in vivo studies documented that OGG1 behaves as a monofunctional DNA glycosylase [44, 45]; thus, catalyzing DNA strand cleavage for the entry of topoisomerase IIβ may need further assistance of APE1.

## The roles of OGG1 in chromatin modifications

**Fig. 4:**
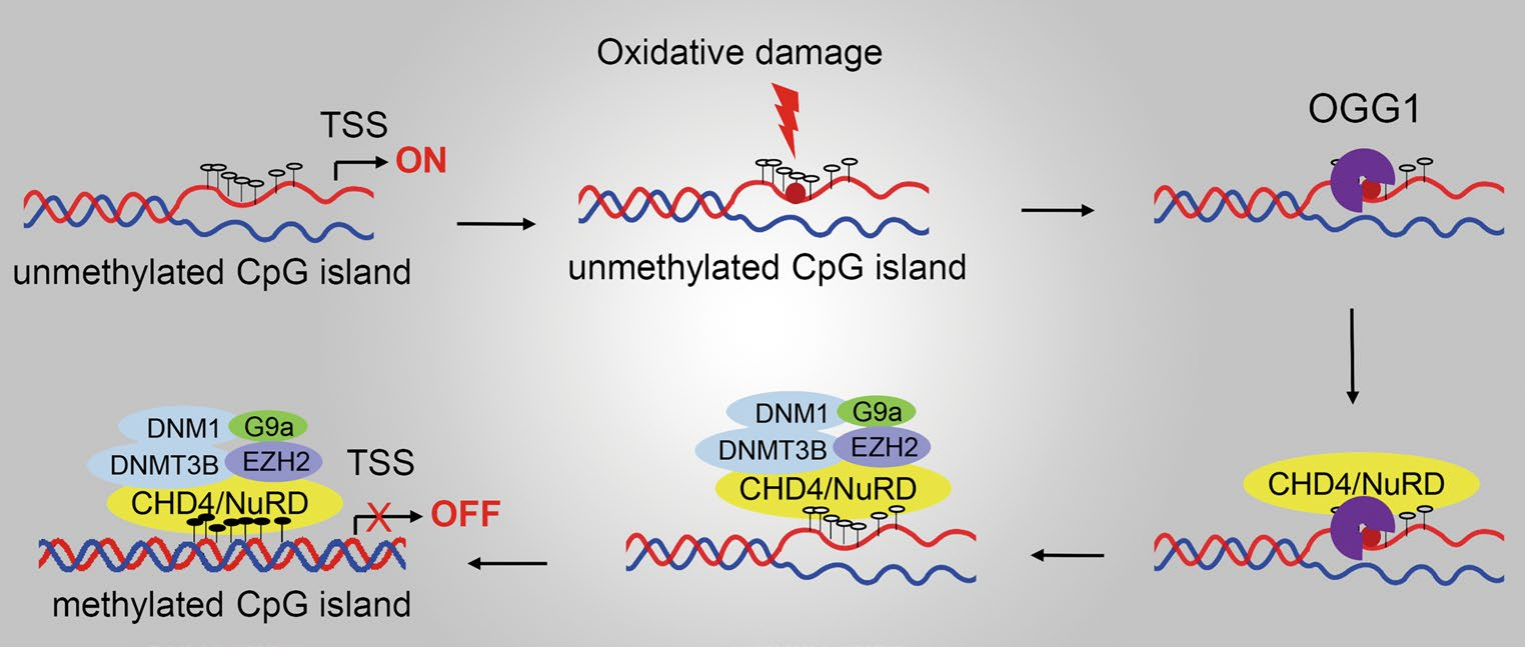
OGG1 recruits chromatin remodelers and modifiers to affect gene expression. CHD4 is recruited by OGG1 to interact with oxidative DNA damage sites. Then CHD4 recruits repressive chromatin proteins including DNA methyl-transferases (DNMT1, 2) and histone H3K27 methyl-transferases (EZH2 and G9a) to DNA damage sites and help to maintain DNA hypermethylation-associated transcriptional silencing of tumor suppressor genes. *CHD4* chromodomain helicase DNA-binding protein 4, *DNMT* DNA methyl-transferase, *EZH2* enhancer of zeste 2 polycomb repressive complex 2, *G9a* euchromatic histone lysine methyl-transferase 2

DNA and histone modifications represent the predominant aspects of epigenetic transcription regulations. A recent study revealed that OGG1 can recruit chromatin remodelers and modifiers to modulate gene expression [62]. Chromodomain helicase DNA-binding protein 4 (CHD4), a component of nucleosome remodeling and deacetylase (NuRD) ATP-dependent remodeling complex, is recruited by OGG1 to oxidative DNA damage sites. Then CHD4 recruits repressive chromatin proteins, including DNA methyl-transferases (DNMTs), enhancer of zeste 2 polycomb repressive complex 2 subunit (EZH2), and euchromatic histone lysine methyl-transferase 2 (EHMT2, also known as G9a) to DNA damage site, where DNMTs impose de novo DNA methylation on cytosines, whereas EHMT2 and G9a catalyze key repressive histone modifications H3K27me3 and H3K9me2, respectively. Repressive chromatin proteins help to maintain transcriptional silencing of tumor suppressor genes [61], which may account for tumorigenesis under chronic oxidative stress (Fig. 4). Importantly, although 8-oxoG accumulates, CHD4 fails to bind with tumor suppressor gene promoter in OGG1-deleted cells, indicating the prior role of OGG1 over 8-oxoG in facilitating the evolution of cancer epigenetic abnormalities.

**Fig. 5:**
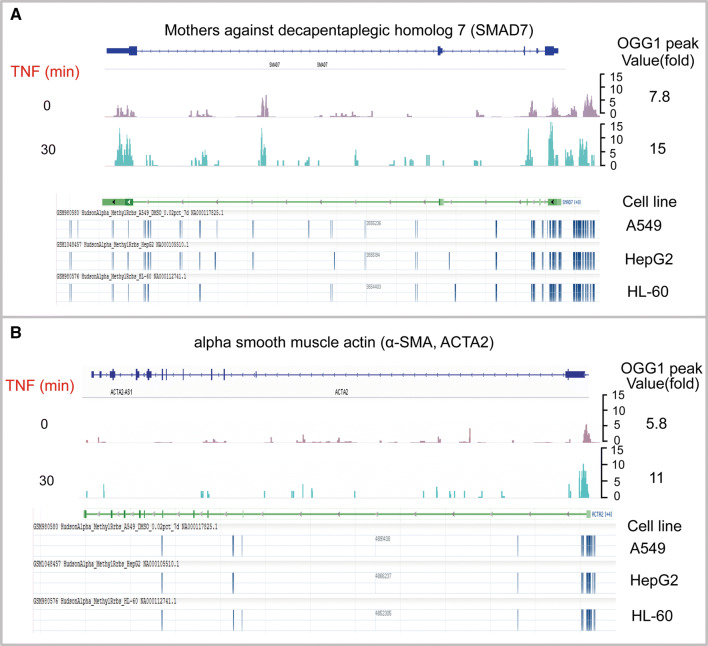
OGG1 peaks are highly correlated with the sites hypermethylated in promoter regions of selected genes. **a** *Smad7* and **b** *α*-*SMA* genes were represented. Upper panels in **a** and **b**, OGG1-ChIP-Seq data (accession #: GSE89017) show the enrichment of OGG1 on indicated genes in cells with or without 30-min treatment of cytokine TNF-α. Images were directly taken from integrative genome viewer (IGV). Lower panels in **a** and **b**, DNA methylation by reduced representation bisulfite-Seq data from ENCODE (accession #: GSE27584) show the methylated sites on indicated genes in A549, HepG2, and HL-60 out of 22 cell lines visualized by genome data viewer

Comparing data between OGG1-ChIP-Seq (accession #: GSE89017) [51] and DNA methylation by reduced representation bisulfite-Seq from ENCODE (accession #: GSE27584) [63], authors of this review also noticed an intriguing correlation between OGG1 peaks and DNA methylation sites in promoter regions of genes, such as those related to tissue remodeling and epithelial–mesenchymal transition (Fig. 5). OGG1 peaks are highly correlated with the sites hypermethylated in *Smad7* and *α*-*SMA* promoters, and importantly, treatment with pro-inflammatory stimuli cytokine TNF-α induced a nearly twofold of increase in OGG1 recruitments. It still requires further investigation to understand the correlation of OGG1 peaks with cytosine methylation sites as well as the implication of inducible increase in OGG1 enrichment to the chromatin imposed by cytosine methylation.

OGG1 binds with promoter-located substrate followed by either recruitment of TFs or chromatin remodelers, suggesting diverse mechanisms of OGG1-mediated modulation of gene transcription. Accumulating studies suggested that OGG1 targets are wide-ranging. Indeed, system analysis of the genomic enrichment of OGG1 by ChIP-seq revealed that OGG1 peaks were primarily located in regulatory regions, especially, guanine-rich promoters; and OGG1-enriched promoters are linked to genes involved in cellular processes such as response to oxidative stress, immune response, signal transduction, and cellular homeostasis [21, 51].

## Post-repair signaling by OGG1·8-oxoG complex

**Fig. 6:**
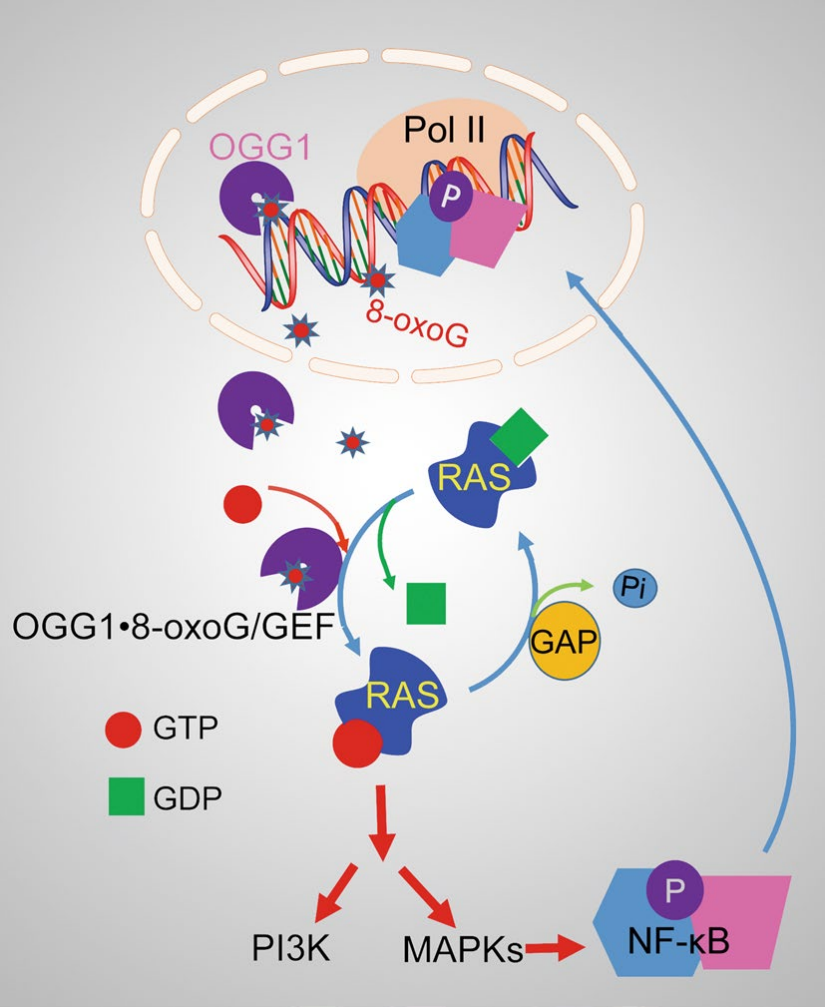
Post-repair signaling by OGG1·8-oxoG complex. A complex of OGG1 with the excised free base (8-oxoG) acts as a guanine-nucleotide exchange factor (GEF) for small GTPases (such as RAS) and thus stimulates signal transduction and activates gene transcription

Promoter-located 8-oxoG plays an epigenetic role in transcription activation; on the other hand, the excised free 8-oxoG base along with its cognate enzyme OGG1 has been shown to induce post-repair cell activation signaling [64–66]. Results from studies in Dr. Boldogh’s laboratory documented the unexpected link between OGG1-initiated BER and cellular signaling via the RAS and RAS homology family GTPases. The role of free 8-oxoG was not obvious until it was observed that OGG1 binds 8-oxoG base (OGG1·8-oxoG complex) with high affinity. FapyG base, a nearly as good substrate for OGG1 when situated in DNA, is not bound by OGG1, and neither is 8-oxoguanosine. This supports the specificity of OGG1 and 8-oxoG base interaction [67]. The implication of these observations became evident from the results, showing that 8-oxoG-induced conformational change in OGG1 allows its interaction with small GTPases. OGG1·8-oxoG complex causes replacement of GDP with GTP in K-RAS, Neuroblastoma RAS viral oncogene homolog (N-RAS), and Harwey-RAS (H-RAS). Follow-up studies documented that OGG1·8-oxoG also catalyzes the GTP → GDP release, so it induces nucleotide releases and allows rebinding [67]; thus, OGG1·8-oxoG functions as a guanine-nucleotide exchange factor (GEF). 8-OxoG base exposure of cells or its *in cellulo* release from genome upon activation of OGG1-BER increases the levels of RAS-GTP. RAS-GTP then induces phosphorylation of cellular homolog of viral raf gene (RAF1), MAPK kinase (MEK1/2), phosphatidylinositol-3-kinases (PI3K), and extracellular signal-regulated kinase (ERK1/2), as well as the nuclear translocation of ERK1/2 [66]. It was reported that the OGG1-initiated repair of genomic 8-oxoG and consequent formation of OGG1·8-oxoG via MAPK and IP3K kinases lead to activation of TFs including NF-κB, increase the expression of pro-inflammatory cytokines/chemokines, and induce robust innate inflammation (Fig. 6). Mice deficient in *Ogg1* showed significantly decreased inflammatory cell recruitment to the airways, whereas a lack of *Nei*-like DNA glycosylases 2 (NEIL2) increased inflammatory responses [66]. RAS activation by 8-oxoG administration was also observed in mouse C2C12 myoblasts, which was followed by activation ERK1/2 and an increase in DNA binding of myogenic regulatory factor D (MyoD) with *Myogenin* promoter. This led to the upregulation of Myogenin and the accumulation of its targets (Dr. Ba’s unpublished data). These data provided the evidence for the hypothesis that exercise-generated ROS could be beneficial for regeneration of adult skeletal muscle [68].

The RAS homology (RHO) family of small GTPases has been shown to interact with OGG1·8-oxoG complex in a guanine-nucleotide-free form, while the Ras-related C3 botulinum toxin substrate 1 (RAC1) interacts with it in GDP-bound form. The latter interaction led to an increased level of RAC1-GTP, but not to GTP → GDP exchange in contrast to H-, K-, or N-RAS. Activation of RAC1 led to increased activity of nuclear membrane-associated NADPH oxidase type 4, consequently to distinct site-specific generation of ROS in the nuclei of cells [69]. These data imply that OGG1-BER → OGG1·8-oxoG → RAC1 activation → site-specific ROS generation is a part of an integrated circuit for genomic 8-oxoG regeneration, which is serving as an epigenetic mark for gene expression.

To examine overall significance of OGG1·8-oxoG complex-driven signaling at system level of gene expression, whole transcriptome analysis was undertaken [70, 71]. Summary of these results allows authors to speculate that OGG1-BER and consequent activation of signaling cascades are links between oxidative stress and post-repair cellular responses, including reestablishment of homeostasis. Indeed, genes activated by OGG1·8-oxoG → RAS/RHO/RAC signaling include ATPases participating in setting electrolyte tone of cells, collagen types, and G protein-coupled receptors—all of which were previously associated with homeostatic states. Another overrepresented biological process is immune system process mediated by C–C and C–X–C cytokines and chemokines—a special composition of mediators that are associated with reestablishment of pre-exposure homeostatic state. These findings lead to a hypothesis that post-repair OGG1·8-oxoG → RAS/RHO/RAC signaling is essential for survival and homeostasis [40, 41]. In addition, OGG1-BER mimicked by repeatedly exposing human diploid lung cells to 8-oxoG base led to G1 cell cycle arrest and pre-matured senescence. High-throughput analysis showed that over 1000 genes were differentially expressed and nearly 90% of genes were identical to those in naturally senesced cells [72]. Gene ontology analysis has identified biological processes driven by small GTPases, PI3Ks, and MAPKs, which led to the hypothesis that chronic OGG1-driven post-repair signaling potentially results in celluar senescence [72]. Taken together, post-repair signaling by OGG1·8-oxoG complex is an important process, the primary function of which is to reestablish pre-exposure cellular/tissue physiological state, but when it is repeated, it may contribute to chronic diseases as well as accelerated aging processes.

## Conclusion

There is mounting evidence, showing that genomic 8-oxoG is not only a pre-mutagenic DNA base lesion but also has an essential role in the modulation of gene expression along with its cognate repair OGG1. As illustrated in Figs. 1, 2, 3, 4, 5, 8-oxoG may serve as an epigenetic mark. OGG1 interacts with the substrates, with or without 8-oxoG excision, inducing conformational changes in adjacent DNA sequences and making easy access of TFs, or recruiting chromatin modifiers/remodelers to their binding sites. Figure 6 shows a second mechanism, which differs from the first by formation of a post-repair complex that is capable of activating small GTPases, and, consequently, downstream cellular signaling. The early studies have linked the accumulation of oxidized guanines to changes in molecular and biological processes, including development of the central nervous and cardiovascular system, Huntington’s disease, obesity-metabolic disorders, mitochondrial dysfunction, and decreased innate and allergic inflammation in OGG1-deficient/*Ogg1* KO mice [73–76]. These epidemiological findings were conventionally explained by genotoxicity of guanine lesions; however, in the view of new evidence, it appears that cells are utilizing 8-oxoG and its cognate repair protein OGG1 to orchestrate a variety of transcriptomes in redox-regulated biological processes. Thus, we may conclude that the deviations/variations from the coordination between the OGG1-initiated repair and transcriptional regulation other than mutagenicity of 8-oxoG account for the etiologic link of 8-oxoG to pathological processes related to immunity, metabolism, cancer or degenerative disorders.

## Abbreviations

8-oxoG: 7,8-Dihydro-8-oxoguanine
APE1: Apurinic/apyrimidinic endonuclease 1
AP site: Apurinic/apyrimidinic site
BER: Base excision repair
CHD4: Chromodomain helicase DNA-binding protein 4
GEF: Guanine-nucleotide exchange factor
HIF-1: Hypoxia-inducible factor-1
KRAS: Kirsten Ras
LSD1: Lysine-specific demethylase 1
NF-κB: Nuclear factor kappa B
OGG1: 8-Oxoguanine DNA glycosylase 1
PQS: Potential G-quadruplex-forming sequences
ROS: Reactive oxygen species
TFs: Transcription factors
VEGF: Vascular endothelial growth factor
